# Detection of micro inclusions in steel sheets using high-frequency ultrasound speckle analysis

**DOI:** 10.1038/s41598-021-99907-4

**Published:** 2021-10-14

**Authors:** Yeonggeun Kim, Jongbeom Kim, Joongho Ahn, Moongyu Han, Hae Gyun Lim, Ki Jong Lee, Juseung Lee, Chulhong Kim, Hyung Ham Kim

**Affiliations:** 1 Department of Convergence IT Engineering, Pohang University of Science and Technology (POSTECH), 77 Cheongam-ro, Nam-gu, Pohang-si, Gyeongbuk 37673 Republic of Korea; 2 Department of Electrical Engineering, Pohang University of Science and Technology (POSTECH), 77 Cheongam-ro, Nam-gu, Pohang-si, Gyeongbuk 37673 Republic of Korea; 3 Department of Mechanical Engineering, Pohang University of Science and Technology (POSTECH), 77 Cheongam-ro, Nam-gu, Pohang-si, Gyeongbuk 37673 Republic of Korea; 4 School of Interdisciplinary Bioscience and Bioengineering, Pohang University of Science and Technology (POSTECH), 77 Cheongam-ro, Nam-gu, Pohang-si, Gyeongbuk 37673 Republic of Korea; 5 Medical Device Innovation Center, Pohang University of Science and Technology (POSTECH), 77 Cheongam-ro, Nam-gu, Pohang-si, Gyeongbuk 37673 Republic of Korea; 6 Department of Biomedical Engineering, Pukyong National University, 45 Yongso-ro, Nam-gu, Busan, 48513 Republic of Korea; 7 Future IT Innovation Laboratory, Pohang University of Science and Technology (POSTECH), 77 Cheongam-ro, Nam-gu, Pohang-si, Gyeongbuk 37673 Republic of Korea; 8 Control and Instrumentation Research Group, Process and Engineering Research Laboratory, POSCO, 6261 Donghaean-ro, Nam-gu, Pohang-si, Gyeongbuk 37859 Republic of Korea

**Keywords:** Imaging techniques, Imaging techniques, Electrical and electronic engineering, Mechanical engineering

## Abstract

With the increasing need for steel sheet quality assurance, the detection of micro-scaled inclusions in steel sheets has become critical. Many techniques have been explored to detect inclusions, e.g., visual inspection, radiography, magnetic testing, and ultrasound. Among these methods, ultrasound (US) is the most commonly used non-destructive testing (NDT) method due to its ease of use and deep penetration depth. However, ultrasound currently cannot be used for detecting the micro-scaled inclusions due to low spatial resolution, e.g., less than 30 μm, which are the key important factors causing the cracks in the high-quality steel sheets. Here, we demonstrate a high-resolution US imaging (USI) using high-frequency US transducers to image micro inclusions in steel sheets. Our system utilizes through-transmission USI and identifies ultrasound scattering produced by the inclusions. We first ultrasonically imaged the artificial flaws induced by the laser on the steel sheet surface for validating the system. We then imaged the real inclusions in the steel sheets formed during manufacturing processes and analyzed them to derive quantitative parameters related to the number of micro-scaled inclusions. Our results confirm that inclusions less than 30 μm can be identified using our high-resolution USI modality and has the potential to be used as an effective tool for quality assurance of the steel sheets.

## Introduction

Steel sheets are widely used for various industrial products, including automotive parts and battery cases^1–3^. These steel sheets withstand large plastic deformation during the manufacturing processes. However, micro-cracks get developed in the steel sheets during this process due to inclusions (e.g., oxides or sulfides). In addition, as the thickness of the target steel sheet becomes thinner and thinner, the size of the inclusion to be managed also becomes smaller^4^. Thus, it is essential to detect these micro-scaled inclusions to produce high-quality steel sheets^5^. More importantly, if the micro inclusions are detected during the manufacturing process, it would significantly save the overall production cost and time, enabling efficient production of high-quality steel sheets.

Various non-destructive testing (NDT) methods have been investigated to detect micro-scaled inclusions. High-resolution visual inspections are widely used due to ease of use^6,7^ but are limited to surface flaws only. Other methods such as thermography, radiography, and magnetic testing (e.g., eddy-current testing and magnetic flux leakage testing) have also been studied to overcome penetration depth limitations. However, thermography requires additional heating to change the tested sample^8^, and radiography uses harmful ionizing radiation^9,10^. The typical magnetic testing approaches can only test ferrous specimens^11,12^, and in particular, eddy-current testing only detects the subsurface defects^13^. In addition, all these methods suffer from low spatial resolution. Thus, the need still exists to develop a high-resolution NDT method to map the inclusions.

The ultrasound (US) NDT has been used widely to detect internal flaws in various industrial products^14–17^. In particular, high-frequency US is used to detect micro-scaled inclusions. For example, a 10-MHz US transducer was used to detect pores varying from several hundred microns to several millimeters and non-metallic inclusions of size smaller than 200 μm^15^. The 25-MHz cylindrically focused US transducers were used to detect the inclusions up to 60 μm × 30 μm^18^. In addition, Chen et al. used 50- and 100-MHz transducers to estimate 50-, 60-, 71-, and 41-μm sized inclusions^19^. However, using these approaches, inclusions smaller than 30 μm could not be detected. Although the detectable sizes of inclusions decreased with high-frequency transducers, it is still challenging to detect the inclusions less than 30 μm, which are the key important factors causing the cracks in the high-quality steel sheets^20–22^. Independent of the frequency of the transducer, it is fundamentally difficult to resolve the inclusions less than 30 μm using the echo signals only due to the low sensitivity and signal-to-noise ratios (SNRs).

Previously, Pandey et al. applied the ultrasonic scattering analysis in US C-scan images to measure the steel cleanliness^23^ using 5- and 10-MHz US transducers. However, inclusion sizes ranged mainly between 31 to 79 μm. Here, we present an improved high-resolution US NDT system using a pair of high-frequency US transducers (e.g., 30 MHz or above) and US scattering analysis. We first validated the system using artificially induced flaws, and then the internal structures of the real steel sheets were ultrasonically delineated. The scattering analysis was performed deriving the number, size, and area of the speckles^24–27^, to evaluate the steel cleanliness. We confirmed that the sizes of the inclusions using the invasive optical microscope and were approximately 20 μm. The results confirm that the high-frequency US NDT imaging modality designed here with scattering analysis has the potential to detect the micro-scaled inclusions and evaluate the steel cleanliness. In the next few subsections, we present our system and algorithm employed to detect the inclusion.

## Methods

### Fabrication of a high-frequency single-element ultrasound transducer

**Figure 1:**
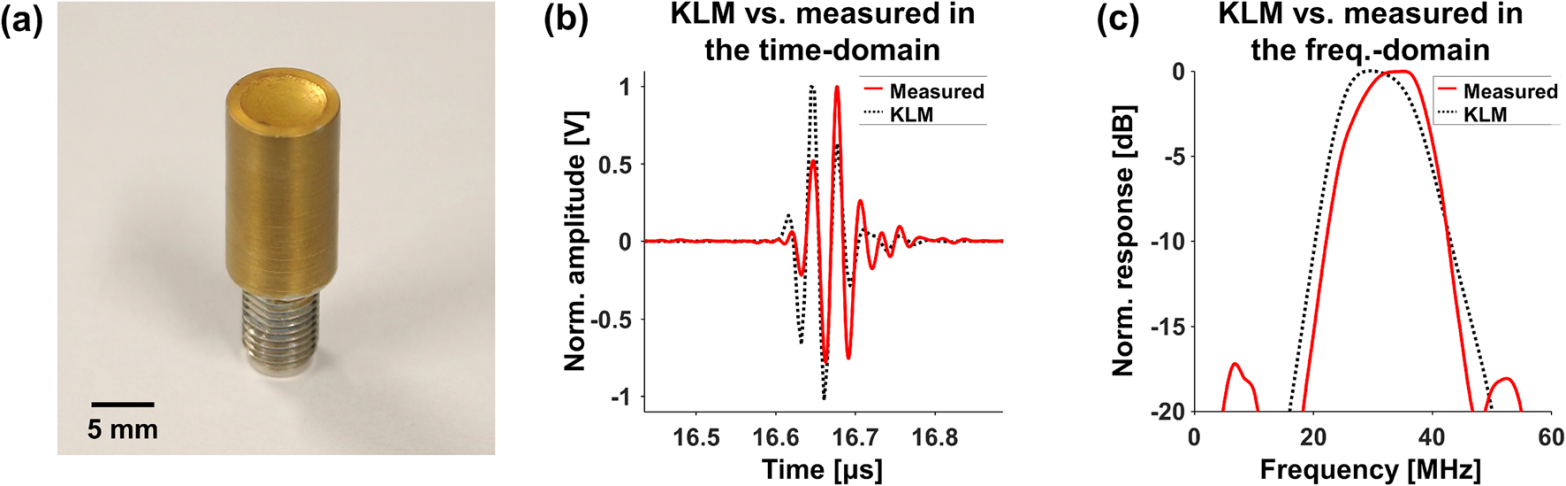
Fabricated US transducer and its specifications. (**a**) Photograph of the fabricated US transducer. Pulse-echo impulse response comparing to the KLM simulation in the (**b**) time-domain and (**c**) frequency-domain. (plotted by: MATLAB R2018a—https://www.mathworks.com/products/matlab.html).

A single-element spherically focused 30-MHz US transducer was fabricated to acquire ultrasonic volumetric data of the steel sheets^28,29^. The following two important steps were addressed in the fabrication of the US transducer: fabricate acoustic stack for transmitting and receiving US pulses, and house the assembly for grounding and protecting. The acoustic stack consists of piezo layer (LiNbO_3_, Boston Piezo-Optics, Bellingham, MA, USA), matching layer (2^–^3 μm silver epoxy mixture, Sigma Aldrich, St. Louis, MO, USA), and backing layer (E-Solder 3022, Von Roll, Schenectady, NY, USA). They were electrically connected by Cr/Au sputtered electrodes. The thickness of each layer and shape of the acoustic stack was optimized through simulation using the Krimholz, Leedom, and Matthaei (KLM) model (PiezoCAD, Sonic Concepts, Bothell, WA, USA)^30^. The simulation result was obtained using the matching layer, piezo layer, and backing layer of thickness 12, 98, and 2000 μm, respectively, and the 6-mm circular aperture shape. The fabricated acoustic stack was fixed to one side of the brass tube by epoxy (Epotek301, Epoxy Technology, Billerica, MA, USA) and wired to the connector (SMA type) on the other side of the brass tube. A 12-mm focal distance was obtained by physically pressing the acoustic stack of the assembled transducer with a 24-mm diameter steel ball. In addition, the length of the focal zone was theoretically calculated to be 196 μm and 784 μm for water and steel, respectively^31^. Finally, the apparatus was coated with Parylene C to protect from water. The photograph of the fabricated US transducer is shown in Fig. 1a.

The specifications of the transducer were theoretically simulated and experimentally measured by a pulse-echo test^32^. A pulser/receiver (DPR500, JSR Corporation, Pittsford, NY, USA) was used to transmit and receive the US pulses through the transducer. The detected US signals were analyzed in both time- and frequency-domains using a computer with a digitizer (ATS9360, Alazar Technologies, Pointe-Claire, QC, Canada) and MATLAB (MathWorks, Natick, MA, USA). The simulation result showed that the center frequency of the transducer to be about 32 MHz, and the − 6-dB bandwidth to be about 56%. The corresponding experiments confirmed the center frequency (31 MHz) and the bandwidth (52%) as shown in Fig. 1b,c.

### High-resolution ultrasound imaging system using raster scanning

**Figure 2:**
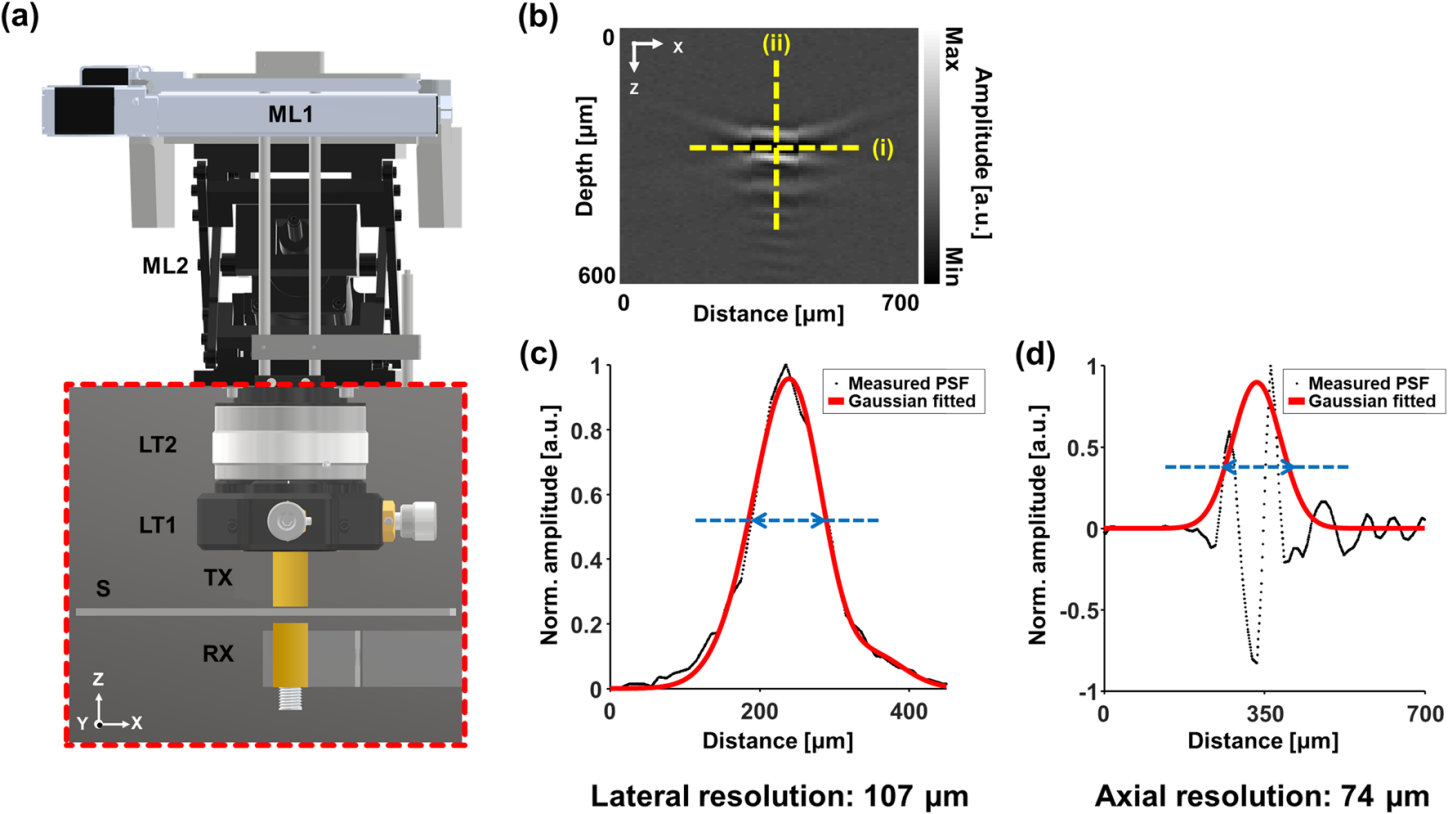
High-resolution ultrasound (US) imaging system. (**a**) System schematic. (**b**) US B-mode image of a steel wire with a diameter of 40 μm. (**c**) Lateral and (**d**) axial profiles cut along the dotted yellow line "i" and "ii", respectively, in (**b**). *ML* motorized linear stage, *LT* linear translator, *TX* transmitting transducer, *RX* receiving transducer, *S* steel sheet, *PSF* point spread function. (plotted by: MATLAB R2018a—https://www.mathworks.com/products/matlab.html, Autodesk Inventor Professional 2020—https://www.autodesk.com/products/inventor/overview?us_oa=dotcom-us&us_si=90f91e34-c852-4086-bf8f-5727edba5268&us_st=inventor&us_pt=INVNTOR, imported by: CXY1Q, Thorlabs Inc.—https://www.thorlabs.com/thorproduct.cfm?partnumber=CXY1Q#ad-image-0, SM1ZM, Thorlabs Inc.—https://www.thorlabs.com/thorproduct.cfm?partnumber=SM1ZM, ASR100B120B-T3A, Zaber Technologies Inc.—https://www.zaber.com/products/scanning-microscope-stages/ASR/documents?part=ASR100B120B-T3A, 8MVT188-20, Standa Ltd.—https://www.standa.lt/products/catalog/motorised_positioners?item=70).

The high-resolution US imaging system using raster scanning is shown in Fig. 2. The developed system was customized for acquiring the US volumetric data of steel sheets (Fig. 2a). The two US transducers faced each other, and their placement enabled both through-transmission and pulse-echo modes. In the through-transmission mode, one transducer transmits the US pulses through the object, and the other transducer receives the through transmitted US waves. In the pulse-echo mode, only one transducer both transmits and receives the US wave. The generation and reception of these US waves were carried out using the pulser/receiver and the digitizer. For precisely aligning the US waves, the transducers were located on an optomechanical XY translation mount (CXY1Q, Thorlabs Inc., Newton, NJ, USA) and a Z-axis mount (SM1ZM, Thorlabs Inc., Newton, NJ, USA). The US module was fixed on a motorized XY microscope stage (ASR100B120B-T3A, Zaber Technologies Inc., Vancouver, BC, Canada) with an accuracy of 40 μm (0.001575″) to acquire the volumetric data. All the above components were installed on a motorized vertical translation stage (8MVT188-20, Standa Ltd., Vilnius, Lithuania). The system was controlled by a data acquisition (DAQ) board (PCIe-6320, National Instruments Corp., Austin, TX, USA) and LabVIEW (National Instruments Corp., Austin, TX, USA). The DAQ board triggers the pulser/receiver, digitizer, and stages for the 2D raster scan. The DAQ board fully synchronized the sequence of acquisition and movement along the X-axis. Integrating the sequence with Y-axis transitions enabled 2D raster scan, and integrating with Z-axis transitions enabled 3D data acquisition.

A steel wire with a diameter of 40 μm was used to measure the spatial resolutions (Fig. 2b–d)^33^. It was placed perpendicular to the beam direction and at the focal point, i.e., horizontal wire positioned at 12.4 mm away from the transducer surface. The steel wire was scanned 10-times with a step size of 10 μm. After scanning the steel wire, the point spread function (PSF) was interpolated with a step size of 1 μm, and a Full Width at Half Maximum (FWHM) of PSF was measured to estimate the spatial resolution. For the lateral resolution (Fig. 2c), the PSF was acquired along the X-direction and Gaussian-fitted. After normalizing the PSF, FWHM was measured. For the axial resolution (Fig. 2d), the PSF was acquired along the Z-axis and was processed similarly. Since the number of pulses used is two or more, envelope-processing using a Hilbert finite impulse response (FIR) filter with a length of 300 was performed before the Gaussian fitting. The lateral resolution achieved was 107 μm, and the axial resolution was 74 μm. The theoretical values for the lateral and axial resolution were calculated to be 98 μm and 46 μm, respectively. We verified that measured values were close to the theoretical values.

### Alignment of ultrasound transducers for through-transmission imaging

**Figure 3:**
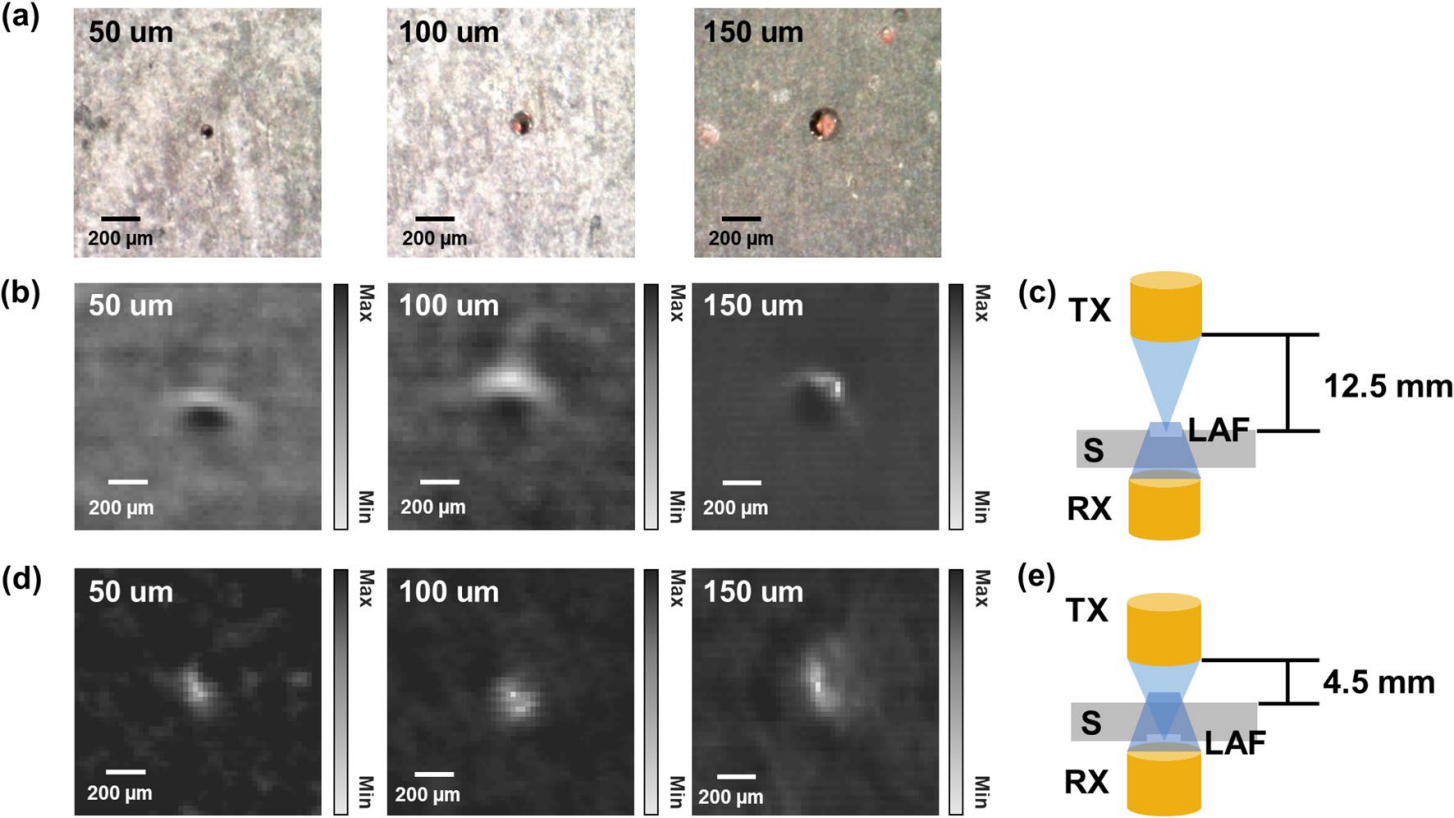
Laser-induced artificial defect (LAF) detection results demonstrating detection of flaws smaller than the lateral resolution of the system. (**a**) The optical microscopic image of the LAFs with a size of 50 μm, 100 μm, and 150 μm. When their position and focus point was at the top surface, (**b**) the US images of the LAFs and (**c**) the diagram. When they were at the bottom, (**d**) the US images of the LAFs and (**e**) the diagram. *TX* transmitting transducer, *RX* receiving transducer, *S* steel sheet, *LAF* laser-induced artificial flaw. (plotted by: MATLAB R2018a—https://www.mathworks.com/products/matlab.html, Microsoft PowerPoint 2016—https://www.microsoft.com/en-us/microsoft-365/powerpoint).

For through-transmission imaging, the placement of transducers and the steel sheet in the axial direction (Fig. 2a) should be determined depending upon the location of the flaws. Since the sound speed in water is different from that in the steel sheet, the distance between the transmitting transducer and the surface of the steel sheet was determined using Eq. (1)

1 $$\mathrm{WP}=\mathrm{F}-\mathrm{MP}\times \left({c}_{steel}/{c}_{water}\right),$$

where WP denotes the water path between the transducer and steel sheet, F is the focal length of the transducer in water, MP the distance to the focal point from the top surface of the steel sheets, c_steel_ and c_water_ is the speed of sound in the steel sheet and water, respectively. The Eq. (1) was calculated with c_steel_ and c_water_ of 5900 m/s and 1480 m/s, respectively^34^. For imaging the laser-induced artificial flaws (LAFs) on the top surface, the transducer was placed at 12.5 mm above the top surface, as shown in Fig. 3c. On the other hand, for imaging the flaw on the bottom surface, the transducer was placed at 4.5 mm, as shown in Fig. 3e. The transducer was moved to 8.5 mm above the steel sheet to focus on the center of the steel sheet to detect the internal flaws. The receiving transducer was placed as close as possible to the steel sheet to minimize the attenuation effect. In all cases, the lateral alignment of the two transducers was conducted by locating the maximum peak of the received waveform.

### Sample preparation and scanning configuration

For our experiments, two sets of steel sheets were prepared. One group was used to verify the echo detection method of the micro-scaled flaws, and the other group was used to verify the scattering analysis method. On the surface of the first group, the artificial flaws were engraved by the laser shots, which enabled controlling the size of the flaw by adjusting the number of repetitions. The diameters of the flaw generated were 50 μm, 100 μm, and 150 μm, as shown in Fig. 3a, and the size of the flaws was checked by confocal microscopy. For each case, 25-mm × 15-mm areas were scanned with a step size of 25 μm.

The other scattering analysis group was categorized into two types: (1) steel sheets with high-density inclusions (SHDI) and (2) steel sheets with low-density inclusions (SLDI). The SHDI and SLDI were classified by quality control specialists in a steel manufacturing site. Two steel sheets were prepared for each SHDI and SLDI (a total of 4 steel sheets). A 25-mm × 25-mm area was scanned twice with a step size of 25 μm for both plates. The US signal along the z-direction was acquired with a step size of 24 μm, and the length of acquired data was equal to the thickness of the steel sheets (2 mm). As a result, a total of 8 3D volumetric data (25 mm × 25 mm × 2 mm) were acquired.

### Image processing and speckle analysis

**Figure 4:**
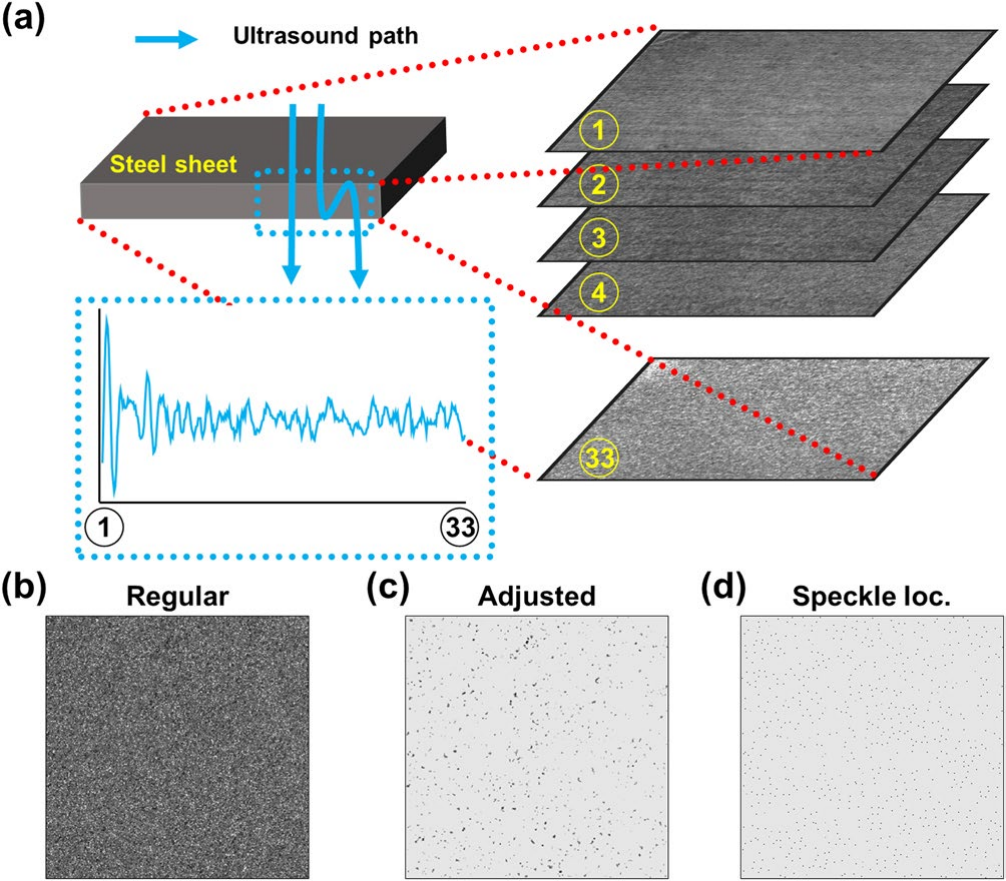
US signal processing procedure. (**a**) Acquisition of 3D volumetric data of the steel sheets and sampling of US C-scan images, (**b**) Acquired US C-scan image after envelope-processing and log-compression, (**c**) US C-scan image after adjusting the threshold, (**d**) US C-scan image after maximum localization of the speckles. (plotted by: MATLAB R2018a—https://www.mathworks.com/products/matlab.html, Microsoft PowerPoint 2016—https://www.microsoft.com/en-us/microsoft-365/powerpoint).

The obtained data from the SHDI and SLDI were demodulated with Hilbert transform and log-compressed to adjust the dynamic range widened by the strong reflection at the boundary between water and steel. The processed data were saved in a 3D array format, setting the transverse plane as US C-scan images in arbitrary (a.u.). The first step in speckle analysis was identifying individual speckles in the US C-scan images. For each volumetric data, 33 US C-scan image planes were selected with a z-step of 120 µm (Fig. 4a), and each plane was cropped to a 17.5-mm × 17.5-mm area to avoid edge artifacts (Fig. 4b). After inverting the C-scan image to grayscale (i.e., the speckles now represented by black spots), the cropped images were first screened using the threshold set by the sum of the mode and the standard deviation of all pixel values in each cropped image. After thresholding, a 3 × 3 median filter was applied to the cropped images to remove noise and one-pixel-sized speckles. We set the filter sizes to 3, which is the minimum size, considering the pixel size of 25 μm. A 2D convolution with zero padding and the stride of 1 was used when applying all the filters. A 3 × 3 Gaussian filter was then used to emphasize the center of the speckles. As a result, the speckles appeared clearly in the processed images (Fig. 4c). Finally, we searched the local maxima to determine the location of the speckles^35^ (Fig. 4d). A pixel was determined as a local maximum point when the intensity of the pixel was higher than that of the adjacent pixels in the horizontal or vertical direction. Adjacent pixels with the same intensity were also defined as the local maximum points.

## Results

### Detection of laser-induced artificial flaws on the steel sheets

The steel sheets with LAFs were scanned using the through-transmission method. In the first scan, the LAFs were placed at the top surface of the steel sheet with the focal point of the transmitting transducer also placed on this surface. Subsequently, the steel sheet was flipped over to place the LAFs at the bottom surface, and the focal point was adjusted accordingly. The scanned data were demodulated with Hilbert transform and log-compressed, and the US C-scan images showing the LAFs were generated. The location of the LAFs in the US image was verified by comparing it with an optical microscope image. The US image was then cropped to a 1.25-mm × 1.25-mm area to extract the individual LAF's local area.

The local images of the LAFs on the top surface (Fig. 3b), as well as the bottom surface (Fig. 3d), are shown in Fig. 3. These images compare the proportional relationship between the shape in US images and the actual size of the LAFs. The detection of LAFs at the bottom surface simulates the detection of the inclusions in a deep area since the US signal needs to penetrate through the steel sheets to detect LAFs at the bottom. The setup would confirm whether our system could detect inclusions not only at the size of the measured lateral resolution (107 μm) but also at smaller ones (50 μm).

In the local image of the LAFs, the amplitude of the US signal from the LAF was higher or lower than in other regions. Whether the US signal amplitude of the LAF was high or low was determined by the location of the US focus. The LAF is hollow with the given diameter (50, 100, or 150 μm), and the depth of the hollow is similar to its diameter. If the US focus was close to the steel sheets surface, the US signal from the surface was higher than LAF. On the other hand, if the US focus was close to the LAF floor, the US signal was lower than the LAF signal. Moreover, in our experiment, the level difference caused by bending of the steel sheet at the location of the flaws was approximately 270 μm. It means that considering the length of the focal zone, i.e., 196 μm in water and 784 μm in steel, the LAF could go out of the focal zone depending on the offset level of the bending. However, the sensitivity and resolution observed (Fig. 3b,d) were acceptable to identify the LAF.

### Speckle analysis result of the steel sheets

The speckle analysis was performed to analyze the scattered signal from the inclusions. The number of the speckles was counted (Table 1) and, SHDI had more speckles than SLDI. For quantitatively comparing speckles of SHDI and SLDI, the size and area of the speckles were calculated based on the location of the speckles. After a 3 × 3 median kernel filtered the cropped image, the intensity profiles along the X- and Y-axis were obtained from each speckle belonging to the median-filtered images, and the two profiles were geometrically averaged. The averaged profiles were Gaussian-fitted, and the FWHM of the profile was considered as the size of the individual speckles. The area was calculated from the local image of 13 × 13 pixels centered on the location of the speckles in the cropped and median-filtered image. The area was assumed to be the number of pixels with pixel values larger than the average of the maximum and minimum pixel values in the local image.

**Table 1:** The result of the number of the speckles at each region (SD: standard deviation).

	Sample number	The number of the speckles		Average	SD
		Region number			
		#1	#2		
SLDI	1	16,945	17,288	17,117	243
	2	14,402	14,570	14,486	119
SHDI	1	18,429	19,139	18,784	502
	2	17,958	19,651	18,805	1197

**Figure 5:**
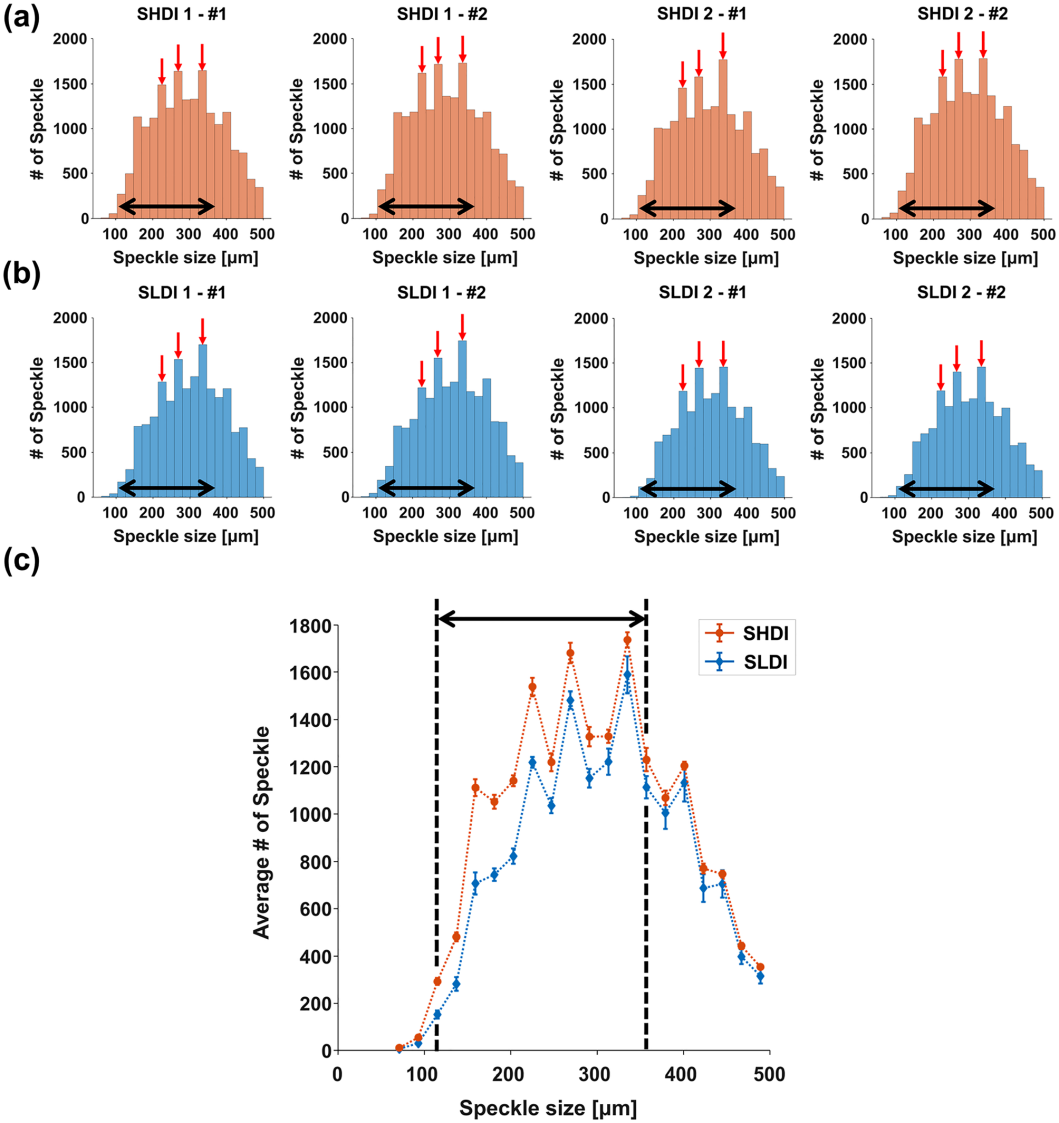
The size histogram results for each region. (**a**) The results of the steel sheets with high-density inclusions (SHDI), and (**b**) low-density inclusions (SLDI). Red arrows indicated the matched peak columns. (**c**) Mean values and standard error of the mean (SEM) of the speckle size for the SHDI and SLDI. Black arrows indicated the region with a greater number of speckles in the SLDI than SHDI. (plotted by: MATLAB R2018a—https://www.mathworks.com/products/matlab.html).

The distributions of the size and area of each speckle were analyzed using a histogram method. In size histograms of SHDI and SLDI, the speckles' size was distributed in a range from 71 to 489 μm (Fig. 5a,b). Interestingly, in the range from 115 to 357 μm (black dotted arrows in Fig. 5), the number of speckles of SHDI is larger than that of SLDI. The means and standard errors of the mean (SEM) for each bin were calculated for the SHDI and SLDI to statistically analyze the speckles in size histograms (Fig. 5c). The statistical result clearly shows the difference between the SHDI and SLDI in this range. The difference implies the scattering signal by the inclusions generated the speckles between 115 and 357 μm.

**Figure 6:**
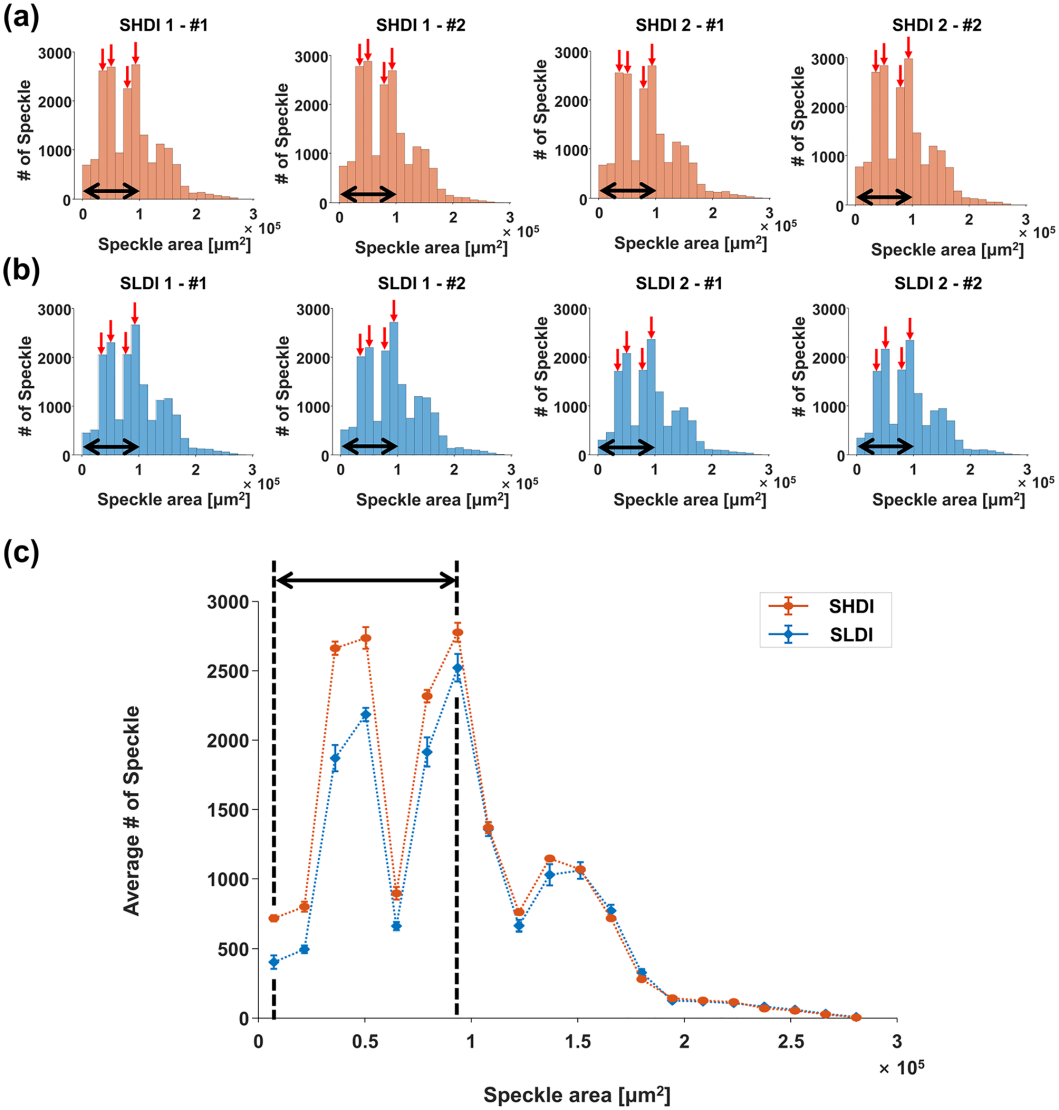
The area histogram results for each region. (**a**) The result of the steel sheets with high-density inclusions (SHDI), and (**b**) low-density inclusions (SLDI). (**c**) Mean values and standard error of the mean (SEM) of the speckle area for the SHDI and SLDI. The implication of red and black arrows is the same as in Fig. 5. (plotted by: MATLAB R2018a—https://www.mathworks.com/products/matlab.html).

The area histograms of SHDI and SLDI have similar trends as size histograms (Fig. 6a,b). The speckles in area histograms are distributed on a range from 7.20 × 10^3^ μm^2^ to 2.81 × 10^5^ μm^2^. The number of speckles less than 9.36 × 10^4^ μm^2^ (black dotted arrows in Fig. 6) are larger in the SHDI than in the SLDI. Since the area calculated using the diameter of 357 μm is 10.0 × 10^4^ μm^2^, the area of 9.36 × 10^4^ μm^2^ corresponds to the size of 357 μm in size histogram. As a result, the distribution of the area histograms agreed with the size histogram.

**Figure 7:**
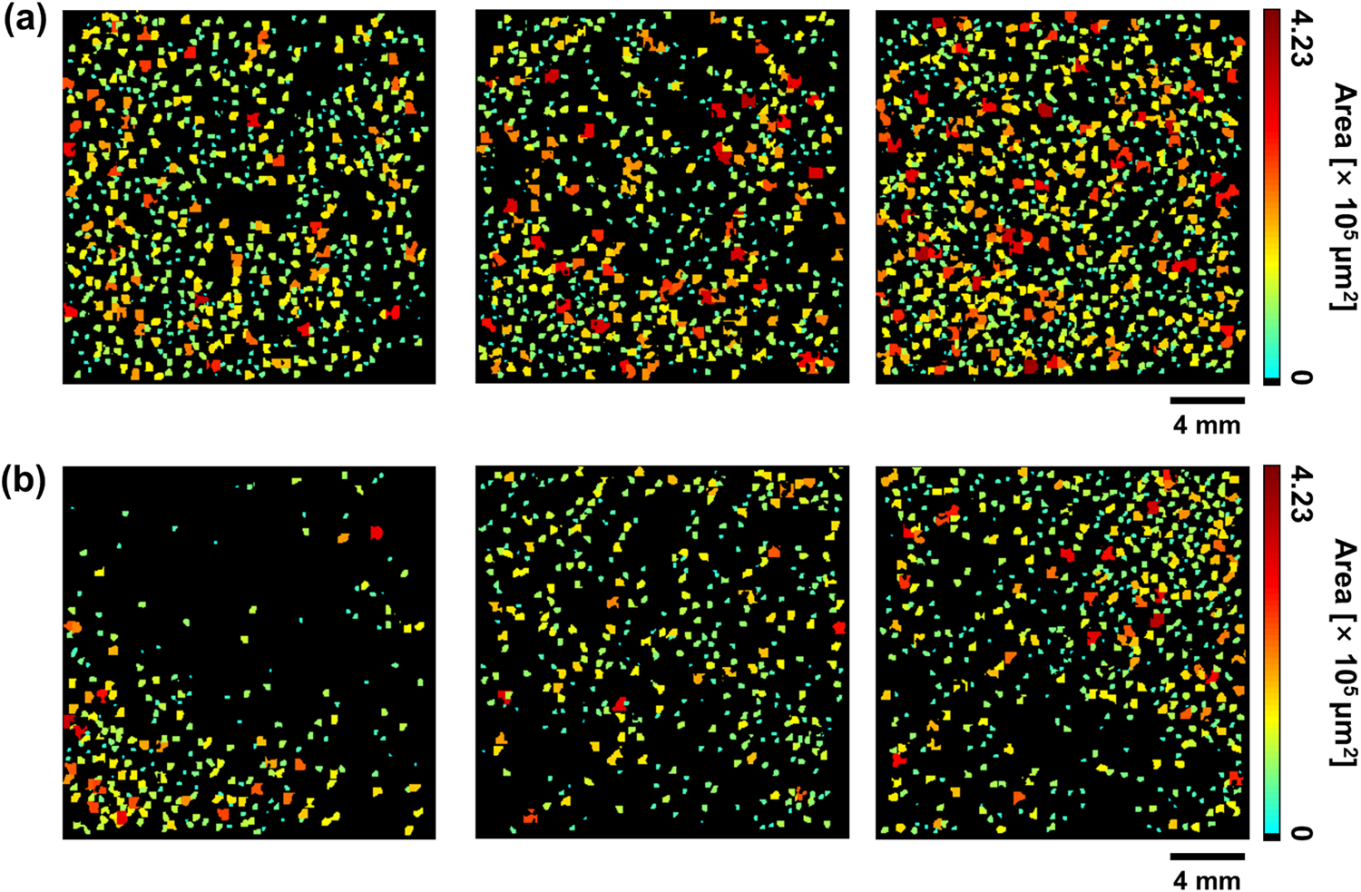
The area map of (**a**) the SHDI and (**b**) SLDI at difference depth. From the left, the area map from the depth above the depth of focus (1/4 depth), in the depth of focus (2/4 depth), and the depth below the depth of focus (3/4 depth). (plotted by: MATLAB R2018a—https://www.mathworks.com/products/matlab.html).

The area maps for all C-scan images showing the distribution of each speckle were plotted. For one scan area of SHDI and SLDI, area maps were extracted from the depth below the depth of focus (DOF), the area above the DOF, and in the DOF (Fig. 7). As a result, area maps of all depths indicated visually that the number of speckles in SHDI is higher compared to that in SLDI. This result validates our speckle analysis on C-scan images obtained at different depths.

### Microscopic observation of the inclusions in steel sheets

**Figure 8:**
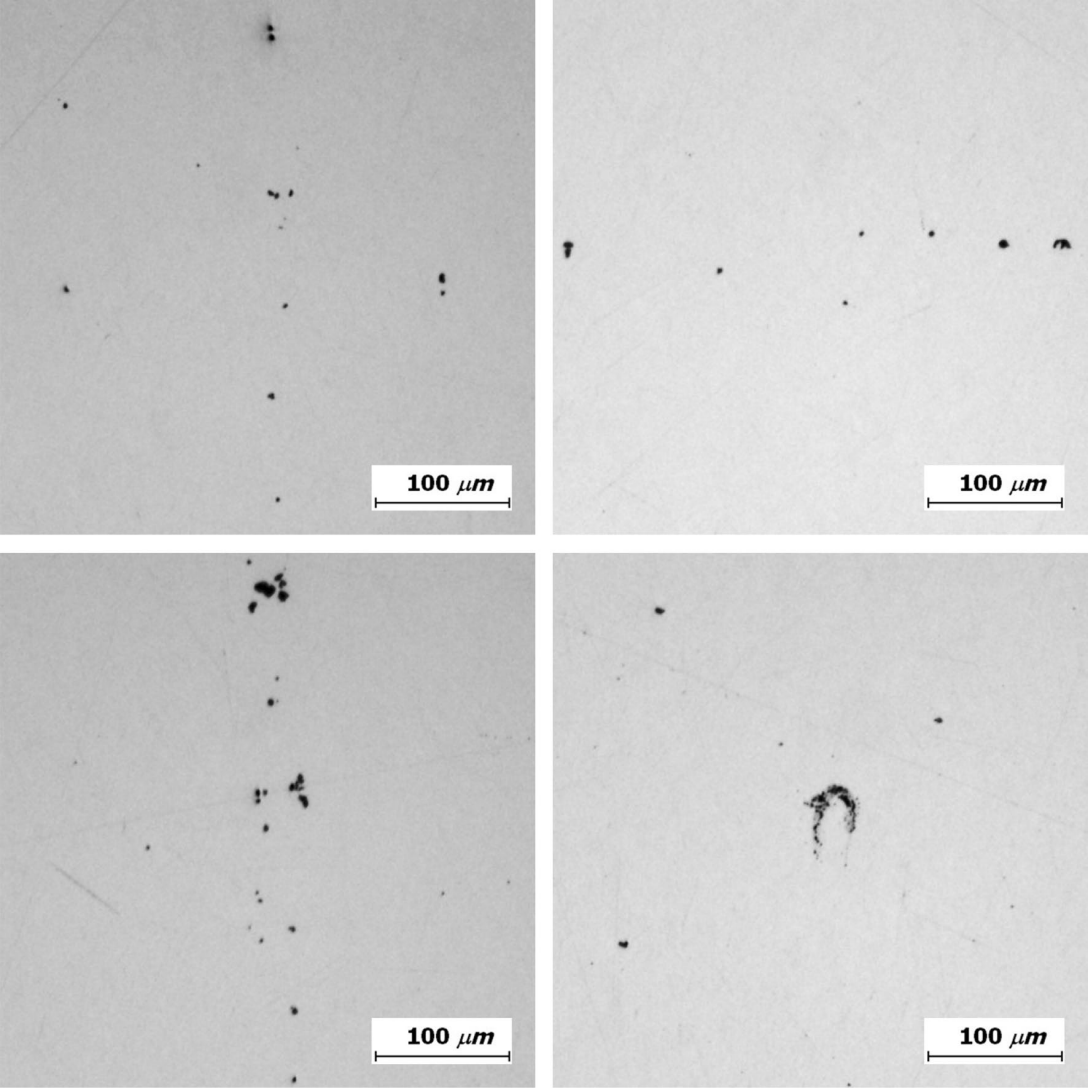
Optical microscopic images of typical inclusions in steel sheets.

Destructive testing was implemented to observe the inclusions directly. After choosing a steel sheet from the SHDI and SLDI, respectively, each steel sheet was cut to 10 mm × 10 mm and molded due to the thin thickness of the steel sheets. The inclusions were exposed at the surface by a series of polishing and observed by optical microscopy (Fig. 8). The cross-section was scanned, and several inclusions were sampled depending on their size and distribution. As a result, the inclusions were observed either alone or in a cluster form and had a size less than 30 μm. In the case of the single form, the size of the observed inclusions was less than 13 μm × 16 μm, and the cluster of the observed inclusions was 34 μm × 42 μm.

## Discussion

We used the through-transmission imaging method to scan the steel sheets in all the experiments. The through-transmission mode has the advantage of obtaining the US C-scan image without distortion resulting from the steel sheet's bending. The pulse-echo imaging method has the challenge of capturing the signal from the same position over multiple experiments due to the irregular distance between the transmitting transducer and steel sheet. However, in through-transmission imaging, the position of the signal is determined by the distance between transmitting and receiving transducers and the thickness of the steel sheets between transducers. Thus, the US signal representing the inclusion of the steel sheet was recorded at the same position by keeping the distance between the plates constant, which enabled acquiring the US C-scan images at the same depth.

Our speckle analysis showed a new parameter estimation technique to evaluate the steel sheets. In all size histograms, common peak values were identified and these values were 225, 269, and 335 μm (red arrows in Fig. 5a,b). The peak values were also identified commonly in all area histogram and these values were 3.55 × 10^4^, 4.97 × 10^4^, 7.81 × 10^4^, and 9.23 × 10^4^ μm^2^ (red arrows in Fig. 6a,b). After calculating the area from the values in size histogram (3.98 × 10^4^, 5.68 × 10^4^, and 8.81 × 10^4^ μm^2^), we found that the peaks in the speckle histograms, which potentially suggest the relation to the grain size. The SHDI and SLDI were identical except for the number of inclusions, which demonstrated that they had the same grain size. Specifically, in the previous research, Zhang et al. used 5- and 10-MHz focused immersion ultrasound transducers on measuring the grain size of the steel using backscattering ultrasonic grain noise^36^. We observe similar findings at the higher frequency of 30 MHz. In this study, we did not address the relationship between speckle size and grain size. However, in our future research, quantitative analysis linking the speckle size and area to the grain size will be addressed. The detection of micro-scale inclusions with the identification of exact location and size is still challenging. One potential solution is to use speckle analysis as suggested here.

The quantitative difference between histograms of the SLDI and SHDI only indicates the presence of inclusions and does not indicate the number of inclusions. After comparing our results with the results of destructive testing, which can show the number of the inclusions, our result can potentially be calibrated to show the number of the inclusions. The calibration between inclusion's size and speckles' size and increasing the frequency or the use of an array-type transducer will be proposed to quantify the findings and improve the resolution of the system.

In summary, we presented an ultrasonic detection of the micro-scaled flaws in steel sheets using two approaches, first using through-transmission imaging of the LAFs on the surface and second using speckle analysis of inclusions. From the imaging of the LAFs, we were able to identify 50-μm sized laser-induced artificial flaws. It proves the feasibility of detecting the inclusions whose size is smaller than the lateral resolution, 107 μm in this case. Next, using speckle analysis of the transmitted US images, we were able to detect the micro-scaled flaws under 30 μm. Further analysis of speckles of the SHDI and SLDI revealed that SLDI presents a lower number of speckles in terms of both its size and area, which is also clearly visible in Fig. 7. Further validation is needed to differentiate the speckles from the grain boundary and those from micro inclusions, but our method can distinguish which of the steel sheets with the same grain size has more inclusions. Thus, we believe the proposed system along with the speckle analysis algorithm, may be used widely to test the quality of the advanced steel sheets. The system can be further enhanced by incorporating new algorithms to quantify the number of inclusions and further improve the resolution with higher frequency transducers.

## Supplementary Information

Supplementary Information.

## Data Availability

*Scientific Reports* requires the inclusion of a data availability statement with all submitted manuscripts, as this journal requires authors to make available materials, data, and associated protocols to readers.
