# Supplementary material for: Detection of micro inclusions in steel sheets using high-frequency ultrasound speckle analysis
Source: Sci Rep. 2021 Oct 14;11:20416. doi: 10.1038/s41598-021-99907-4 (PMC8516875; doi:10.1038/s41598-021-99907-4)
Supplement: Supplementary file 1 — Supplementary Information. [file 41598_2021_99907_MOESM1_ESM.docx]

**Supplementary Information**

**Detection of micro inclusions in steel sheets using high-frequency ultrasound speckle analysis**

**Yeonggeun Kim^1†^, Jongbeom Kim^1†^, Joongho Ahn^1†^, Moongyu Han^1^, Hae Gyun Lim^6^, Ki Jong Lee^7^, Juseung Lee^8*^, Chulhong Kim^1,2,3,4,5*^, and Hyung Ham Kim^1,2,4,5*^**

^1^Department of Convergence IT Engineering, Pohang University of Science and Technology (POSTECH), 77 Cheongam-ro, Nam-gu, Pohang-si, Gyeongbuk, 37673 Republic of Korea

^2^Department of Electrical Engineering, Pohang University of Science and Technology (POSTECH), 77 Cheongam-ro, Nam-gu, Pohang-si, Gyeongbuk, 37673 Republic of Korea

^3^Department of Mechanical Engineering, Pohang University of Science and Technology (POSTECH), 77 Cheongam-ro, Nam-gu, Pohang-si, Gyeongbuk, 37673 Republic of Korea

^4^School of Interdisciplinary Bioscience and Bioengineering, Pohang University of Science and Technology (POSTECH), 77 Cheongam-ro, Nam-gu, Pohang-si, Gyeongbuk, 37673 Republic of Korea

^5^Medical Device Innovation Center, Pohang University of Science and Technology (POSTECH), 77 Cheongam-ro, Nam-gu, Pohang-si, Gyeongbuk, 37673 Republic of Korea

^6^Department of Biomedical Engineering, Pukyong National University, 45 Yongso-ro, Nam-gu, Busan, 48513 Republic of Korea

^7^Future IT Innovation Laboratory, Pohang University of Science and Technology (POSTECH), 77 Cheongam-ro, Nam-gu, Pohang-si, Gyeongbuk, 37673 Republic of Korea

^8^Control and Instrumentation Research Group, POSCO, 6261 Donghaean-ro, Nam-gu, Pohang-si, Gyeongbuk, 37859 Republic of Korea
^*^[davidkim@postech.ac.kr](mailto:davidkim@postech.ac.kr), [chulhong@postech.edu](mailto:chulhong@postech.edu), [dr_leejs@posco.com](mailto:dr_leejs@posco.com)
^⸸^These authors contributed equally to this work.

**Supplementary Information Contents**

- **Supplementary Figure S1:** Bending surface of the steel sheet.
- **Supplementary Figure S2:** The example of original and processed images.
- **Supplementary Table ST1:** The measured size of the LAF in the respective US images.


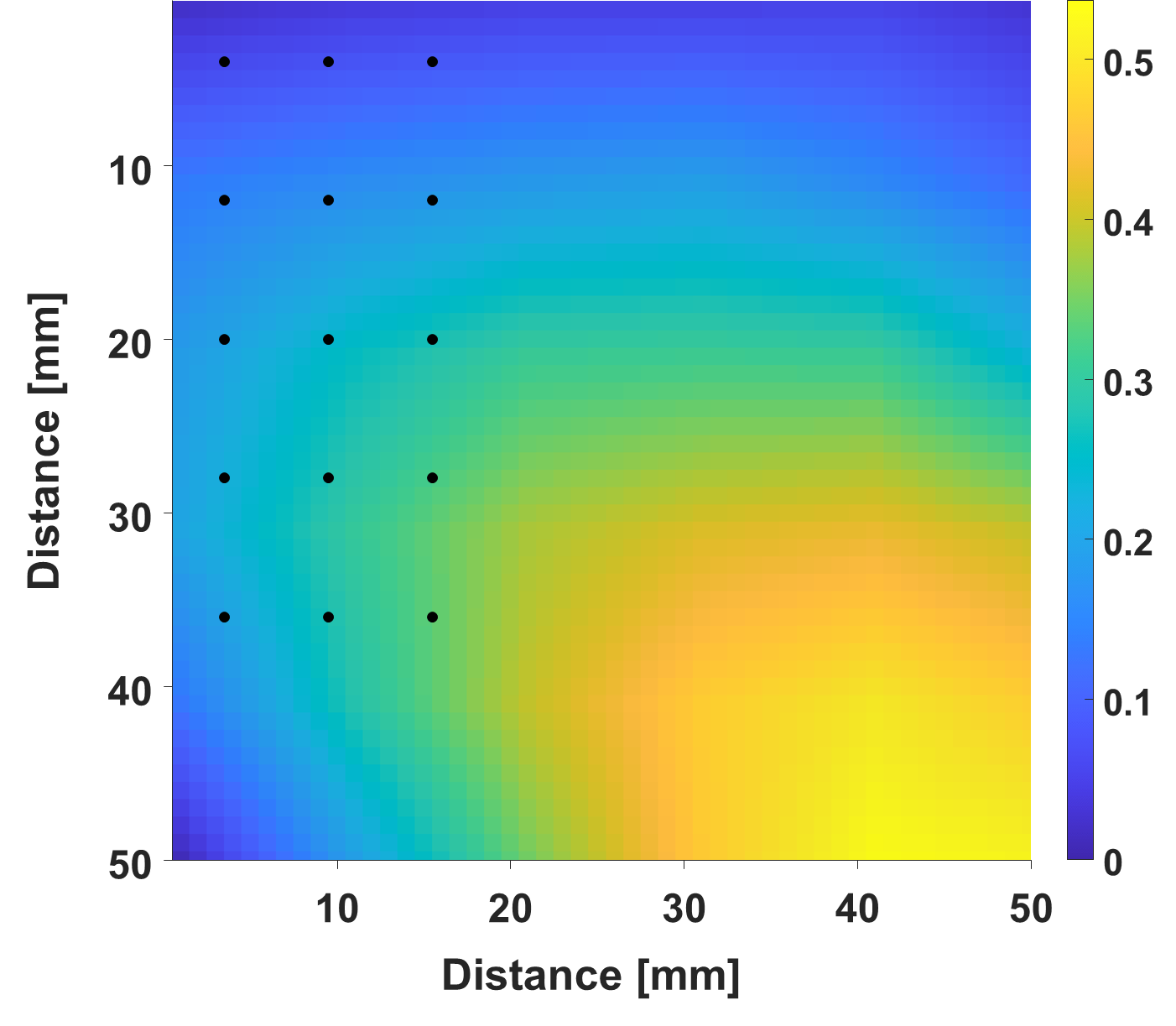


**Supplementary Figure S1.** Bending surface of the steel sheet. The black dots indicated the position of the flaws. Assuming that the steel sheet has a uniform thickness, the local height of the steel sheet was measured. As a result, the maximum difference of steel sheet was 540 μm. Especially, at the location of the flaws, the maximum difference was about 270 μm. (**plotted by**: MATLAB R2018a - <https://www.mathworks.com/products/matlab.html>)


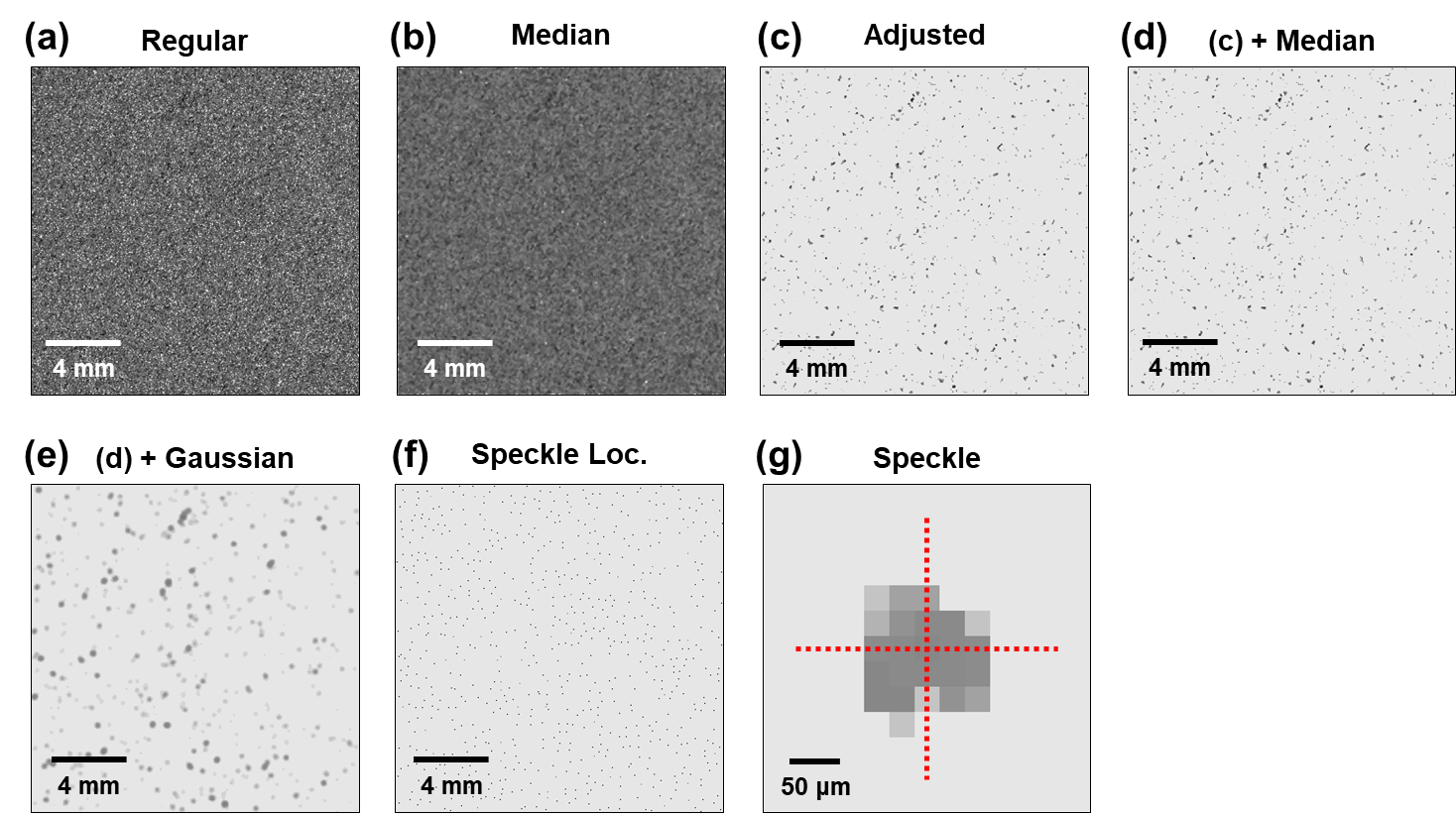


**Supplementary Figure S2.** The example of original and processed images. (a) Cropped image to 17.5 mm × 17.5 mm. (b) Median-filtered image. (c) Adjusted image by the sum of the mode and the standard deviation of all pixel values in each cropped image. (d) Median-filtered image with 3 × 3 median kernel. (e) Gaussian-filtered image with 3 ×3 Gaussian kernel. (f) Speckle localized image. (g) Local image of the speckle. (**plotted by**: MATLAB R2018a - <https://www.mathworks.com/products/matlab.html>)

| The position of the flaws | The size of the LAF | | |
| --- | --- | --- | --- |
|  | 50 μm | 100 μm | 150 μm |
| Top surface | 121 μm | 138 μm | 166 μm |
| Bottom surface | 133 μm | 135 μm | 160 μm |

**Supplementary Table ST1.** The measured size of the LAF in the respective US images.
